# The Importance of Intestinal Length in Triglyceride Metabolism and in Predicting the Outcomes of Comorbidities in Laparoscopic Roux-en-Y Gastric Bypass—a Narrative Review

**DOI:** 10.1007/s11695-021-05421-x

**Published:** 2021-04-29

**Authors:** Pirjo Käkelä, Tuomo Rantanen, Kirsi A. Virtanen

**Affiliations:** 1 Department of Surgery, University of Eastern Finland and Kuopio University Hospital, Kuopio, Finland; 2 Department of Public Health and Clinical Nutrition, University of Eastern Finland, Kuopio, Finland; 3 Department of Endocrinology and Clinical Nutrition, Kuopio University Hospital, Kuopio, Finland

**Keywords:** Obesity, Gastric bypass, Comorbidity, Triglycerides, Weight Loss, Intestine

## Abstract

In this narrative review, we will appraise if modification of the length of bypassed small intestine based on measured total small intestinal length could optimize the outcomes of the laparoscopic Roux-en-Y gastric bypass (LRYGB). We provide a summary of carefully selected studies to serve as examples and to draw tentative conclusions of the effects of LRYGB on remission of comorbidities. As the heterogeneity of the included studies varied in terms of outcomes, type of study, length of the bypassed small intestine, and the follow-up, a common endpoint could not be defined for this narrative article. To achieve efficient metabolic outcomes, it is important to carefully choose the small intestine length excluded from the food passage suited best to each individual patient.

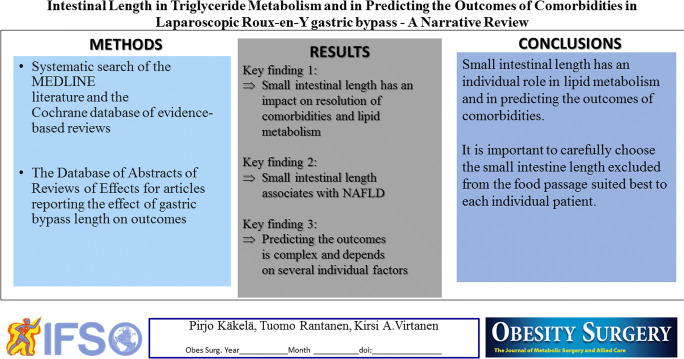

## Introduction

Globally, obesity is affecting more than 600 million people and the trend is upward. More than half a million obese people are admitted to obesity surgery each year [1]. As a result, significant numbers of people are living with a new anatomical condition. In 2019, the IFSO Global Registry amalgamated data from 61 different countries. About 58% of operations were laparoscopic sleeve gastrectomies [1].

Recommendations for bariatric surgery are changing worldwide. In 1991, the National Institutes of Health (NIH) Consensus Development Panel gave a significant number of recommendations for bariatric surgery [2]. These recommendations are a majority consensus, rather than evidence based and they are accepted with very minor variations in most western countries. Same recommendations are used in Finland and worldwide. Indications for bariatric surgery are a body mass index (BMI) of ≥ 35 kg/m^2^ with at least one comorbid condition or a BMI of ≥ 40 kg/m^2^. Age limits are set in Finland between 18 and 65 years, but an individual evaluation is possible [3].

**Fig. 1:**
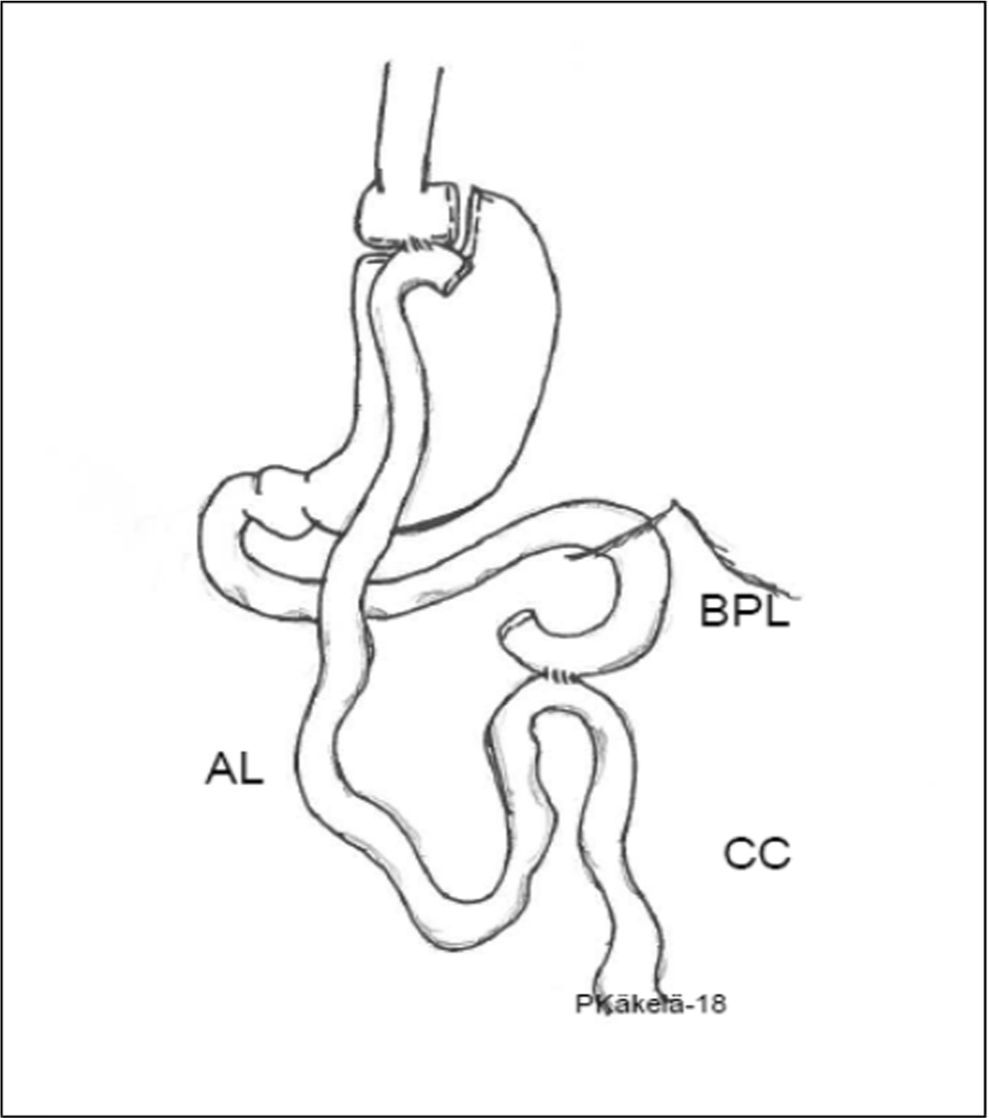
Roux-en-Y gastric bypass

In 2011, the International Diabetes Federation recommended bariatric surgery to patients with a BMI between 30 and 35 kg/m^2^ who, regardless of weight loss and conventional medical therapy, have uncontrolled diabetes [4]. The National Institute for Health and Care Excellence and the American Diabetes Association followed similar recommendations [5]. Also, in Finland, the Current Care Guidelines was recently recommended in the same way [3]. These new guidelines increase significantly the need for bariatric surgery. Weight loss is recommended for all obesity-related diseases, but weight loss programs with conventional therapy are successful only for a fraction of obese people, and the long-term results are very modest [6]. Sleeve gastrectomy is the most frequently performed procedure. However, LRYGB is highly effective hormonal procedure for weight loss and for resolution of comorbidities [7]. In recent years, the role of different procedures has been revised following the postoperative outcomes from a metabolic and functional point of view. The superiority of bariatric surgery is recognized worldwide as a cost-effective treatment both in terms of weight loss, maintenance of weight loss, and remission of comorbidities, such as type 2 diabetes (T2DM), hypertension, hyperlipidemia, and non-alcoholic fatty liver disease (NAFLD) [8, 9]. Sixty years after the introduction of the laparoscopic Roux-en-Y gastric bypass (LRYGB) by Mason and Ito [10], there is no consensus on the ideal length of the gastric bypass limbs. Much variability exists among surgeons even for similar patient BMIs. In literature, the reported length of the alimentary limb (AL) and biliopancreatic limb (BPL) ranges widely among surgeons (average 110 (range 35–250) cm and 48 (range 10–250) cm, respectively) [11] (Fig. 1). Some authors believe that the limb length currently used makes the LRYGB a mainly restrictive rather than malabsorptive procedure.

## Methods

We systematically searched the MEDLINE literature, limited to English language articles. Using the same strategy, we searched the Cochrane database of evidence-based reviews and the Database of Abstracts of Reviews of Effects for articles reporting the effect of gastric bypass length on outcomes.

## Results

LRYGB is the most frequently executed restrictive, malabsorptive [7], and hormonal [12] operation in Europe and in Latin America [1]. LRYGB is a gastric bypass featuring a gastric pouch, a gastro-jejunostomy and a jejuno-jejunostomy (Fig. 1). As a result of this configuration, absorption of nutrients occurs mostly distal to the jejuno-jejunostomy, when food particles interact with the digestive pancreatic enzymes. Thus, the length of functionally absorptive small intestine is decreased by 160–225 cm as a result of the operation. The degree of malabsorption can be modified by altering the length of these limbs. As a result, food is diverted from the small gastric pouch directly into the jejunum and bypassing the gastric remnant, duodenum, and proximal jejunum. However, according to DUCATI trial, some absorption of amino acids and glucose may also take place in the AL as well, through the saliva and succus which may digest proteins and carbohydrates [13].

When altering the gastrointestinal anatomy and physiology, the amount of food intake is reduced and nutrients are delivered more rapidly to the distal small intestine. Patients will lose weight, and they will also have favorable metabolic improvements due to the modifications in the entero-endocrine axis (e.g., increase of peptide YY (PYY), glucagon-like peptide-1 (GLP-1) levels) [14–16]. This all can lead to a loss of hunger and increase in satiety [17]. Exclusion of the proximal small intestine reduces the secretion of upper gastrointestinal factors such as anti-incretins, which are assumed to suppress insulin secretion or promote insulin resistance [12]. Changes in gut microbiota after the bypass may also influence weight by reduction of low-grade inflammation associated with obesity [18].

The length of the common channel (CC) is not routinely measured during LRYGB, and therefore, the length of the total small intestine remains unknown. In addition, the flexible elasticity of bowel makes its measurement sometimes rather subjective and variable and there is no recommended standard method for intestinal measurement. Currently, the data of the normal length of the total small intestine are mostly based on old cadaver studies [19–21]. Very few studies compare the ratio of the AL+BPL to the length of the CC and their impact on resolution of comorbidities. A few publications have shown that after LRYGB, a short CC, which is 100 cm or less, leads to a resolution of more than 95% of comorbidities with 65% excess weight loss (%EWL), and this has a better outcome than a long CC, more than 100 cm [22, 23]. When the length of the CC approaches 100 cm, a significant impact on comorbidities and lipid metabolism is observed due to malabsorption [22]. According to the study of Käkelä et al., along with changes in lipid metabolism, a difference in liver steatosis between groups with different baseline small intestinal length has been found, suggesting that the small intestinal length also associates with NAFLD [24]. An inadequate bypass with a long CC may fail to reach its malabsorptive goal [25, 26], while a too short AL and CC may drive the patient to protein-calorie malnutrition in up to 20–25% of LRYGB procedures [27]. Individual total small intestinal length may be important in predicting the weight loss and resolution of metabolic comorbidities [22, 23]. Measuring the entire small intestine before LRYGB and tailoring the small intestinal limbs according to the BMI may prevent the risk of nutritional consequences in malabsorptive, revisional, and metabolic procedures [28]. During the first 12 weeks following obesity surgery, T2DM control may be achieved in 93% of patients with a short CC (1/3 of the total length of the small bowel) and in 58% of patients with a long CC (2/3 of the total length of the small bowel) [23]. In distal bypass with a short, 100 cm CC, resolution of T2DM reached 94% at the 4-year follow-up [29].

Small intestinal length, gender, and age may be strong predictors of weight [28]. Older women have a shorter small intestine than older men [30]. A shorter small intestine could predispose to weight loss. Men have a longer small bowel and a larger body mass, and these could predispose to weight gain. Further, older people have difficulties to lose weight [28]. According to Nordgren et al., increased weight is positively associated with the small intestinal length [31]. In the contrary, we found that the small intestinal length was longer among women, despite of age [24], and that the weight loss was equal, despite the length of the CC, as reported before [32]. However, there is conflicting data reporting that age does not correlate with the small intestinal length [28, 33] and patients with increased weight do not have longer small intestine [30].

A range of 100–200 cm for combined length of BPL or AL gives optimum results with LRYGB in most patients [26]. A long AL of more than one-third of the length of the total small intestine predicts both early and 5-year follow-up weight loss outcomes in superobese (BMI ≥ 50 kg/m^2^) but not in morbidly obese (BMI ≤ 50 kg/m^2^) patients [25]. Choban et al. prefer an AL of approximately one-half of the length of the total small intestine [34]. For patients with a BMI ≤ 50 kg/m^2^, limb lengths are not as crucial to successful weight loss [22]. A systematic review by Mahawar et al. [26], a study by Navez et al. [35], and the DUCATI trial of 444 patients [13] all agree that lengthening of the AL and shortening the CC do not seem to have an effect on the weight loss at short time follow-up. However, further reduction in the length of the CC may lead to an increased incidence of metabolic and nutritional complications [34]. Mahawar et al. found that malabsorption makes only a minor overall contribution of approximately 11.0% to weight loss after LRYGB [36]. The ratio of the BPL to the total length of the small intestine may be crucial. In patients with BMI ≥ 60 kg/m^2^, a ratio of > 45% was associated with higher %EWL at 2 and 3 years [37]. In patients with BMI ≤ 60 kg/m^2^, the benefits of a longer BPL diminished during long-term follow-up [37]. The %EWL was faster with the short CC but was similar in both groups at 48 months (70% vs 74%) [29].

Most of the obesity-related changes in lipid metabolism have been associated with NAFLD [38]. Most importantly, hypertriglyceridemia in obese individuals is closely associated with NAFLD [39]. In addition, cholesterol synthesis in the liver is increased in NAFLD [40, 41]. Accordingly, bariatric procedures produce significant improvements in NAFLD and levels of serum lipids, but the response varies widely due to anatomic alterations unique to each bariatric procedure [42]. Some studies suggest a better control of dyslipidemia with a short CC, because of increased lipid malabsorption [23]. It is not known if the different small intestinal length is associated with NAFLD and lipid metabolism and how much the response in serum lipids can be modified by different lengths of CC. Pinheiro et al. found out that dyslipidemia was improved in 70% of patients with a short CC (1/3 of the total length of the small bowel), whereas only 57% of the patients with a long CC (2/3 of the total length of the small bowel) showed improvement in dyslipidemia [23]. Nelson et al. found that dyslipidemia was resolved in 68% of patients with a short CC, whereas only 44% of the patients with a long CC showed improvement [43]. In contrast, Valera-Mora et al. suggested that changes in serum lipids in response to surgery are independent of the length of the CC [44]. According to the study of Käkelä et al., hyperlipidemia improved in about 70% of patients after surgery [24] which is in line with previous studies [23, 45]. Also, serum triglyceride (TG) levels associated with the small intestinal length before and the length of the CC after LRYGB [24], suggesting that the small intestinal length regulates lipid metabolism, as published before [44, 46, 47]. It is important to note that CC length after surgery was variable between the study groups, while the length of the AL and BPL was the same in every group [24]. Therefore, the differences between the study groups after the surgery are likely to be due to different lengths of the CC. There are also several other studies that have found that the CC length associates with more than 95% resolution or improvement in obesity-related comorbidities including hyperlipidemia [22, 44, 46–48].

## Discussion

Bariatric surgery is an effective long-term treatment for severe obesity that results in long-term weight loss; improvement and remission of obesity-related comorbidities, particularly T2DM, dyslipidemia, and NAFLD; improvement in quality of life; and prolonged survival. Individual total small intestinal length may be important in predicting the weight loss and resolution of metabolic comorbidities.

The fact that the length of the AL is of limited relevance to postoperative weight loss for the patients with BMI ≤ 50 kg/m^2^ but may make a difference for the superobese (BMI ≥ 50 kg/m^2^) is not surprising. The evidence from the distal gastric bypass and biliopancreatic diversion literature is clear. The degree of malabsorption is dependent on the length of the CC and the ratio of the bypassed AL+BPL. If malabsorption and improvement in dyslipidemia is a primary goal of the gastric bypass, preoperative measurements should focus on the length of the CC, rather than the AL and the BPL. However, current clinical evidence suggests that malabsorption is not as important as it was thought of in the past, with regard to both weight loss and improvement of lipid metabolism. On the contrary, these features are mediated primarily by gut-related hormonal processes [14–16]. Limitations of the available literature include variation in the length of the small intestinal limbs, which makes comparison of results between the studies challenging. Some studies consider the same AL or BPL short while the others long. Furthermore, the criteria used to determine the length of the limbs vary significantly among surgeons [48].

Together, these findings suggest that not only the small intestinal length regulates lipid absorption, but also gut-related hormones after obesity surgery. However, modification of the length of bypassed small intestine based on measured total small intestinal length might optimize the outcomes of the elective LRYGB. Additionally, Käkelä et al. suggest that instead of constructing the standard bypass, the ratio of the bypassed AL+BPL vs CC length should be 2/3 vs 1/3 of the total length of the small bowel when better control of dyslipidemia is aimed for [24]. The exact mechanism remains open but could be related to alterations in lipid absorption. This requires that the length of the total small intestine is measured preoperatively when constructing a gastric bypass, the procedure we have systematically been performing. However, we must pay attention to the possibility of inadvertent injury of the small intestine when measuring it.

## Conclusion

The question, “how important the intestinal length is in triglyceride metabolism and in predicting the outcomes of comorbidities in LRYGB?”, cannot be answered in definitive terms but must be answered for each patient individually, as it is complex and depends on several individual factors, such as comorbidities and preoperative weight.
